# Stress Survival Islet 2, Predominantly Present in Listeria monocytogenes Strains of Sequence Type 121, Is Involved in the Alkaline and Oxidative Stress Responses

**DOI:** 10.1128/AEM.00827-17

**Published:** 2017-08-01

**Authors:** Eva Harter, Eva Maria Wagner, Andreas Zaiser, Sabrina Halecker, Martin Wagner, Kathrin Rychli

**Affiliations:** Institute for Milk Hygiene, Milk Technology and Food Science, University of Veterinary Medicine Vienna, Vienna, Austria; University of Bayreuth

**Keywords:** Listeria monocytogenes, genetic hypervariable hot spot, genetic islet, hotspot, persistence, stress response

## Abstract

The foodborne pathogen Listeria monocytogenes is able to survive a variety of stress conditions leading to the colonization of different niches like the food processing environment. This study focuses on the hypervariable genetic hot spot *lmo0443* to *lmo0449* haboring three inserts: the stress survival islet 1 (SSI-1), the single-gene insert *LMOf2365_0481*, and two homologous genes of the nonpathogenic species Listeria innocua: *lin0464*, coding for a putative transcriptional regulator, and *lin0465*, encoding an intracellular PfpI protease. Our prevalence study revealed a different distribution of the inserts between human and food-associated isolates. The *lin0464-lin0465* insert was predominantly found in food-associated strains of sequence type 121 (ST121). Functional characterization of this insert showed that the putative PfpI protease Lin0465 is involved in alkaline and oxidative stress responses but not in acidic, gastric, heat, cold, osmotic, and antibiotic stresses. In parallel, deletion of *lin0464* decreased survival under alkaline and oxidative stresses. The expression of both genes increased significantly under oxidative stress conditions independently of the alternative sigma factor σ^B^. Furthermore, we showed that the expression of the protease gene *lin0465* is regulated by the transcription factor *lin0464* under stress conditions, suggesting that *lin0464* and *lin0465* form a functional unit. In conclusion, we identified a novel stress survival islet 2 (SSI-2), predominantly present in L. monocytogenes ST121 strains, beneficial for survival under alkaline and oxidative stresses, potentially supporting adaptation and persistence of L. monocytogenes in food processing environments.

**IMPORTANCE** Listeria monocytogenes strains of ST121 are known to persist for months and even years in food processing environments, thereby increasing the risk of food contamination and listeriosis. However, the molecular mechanism underlying this remarkable niche-specific adaptation is still unknown. Here, we demonstrate that the genomic islet SSI-2, predominantly present in L. monocytogenes ST121 strains, is beneficial for survival under alkaline and oxidative stress conditions, which are routinely encountered in food processing environments. Our findings suggest that SSI-2 is part of a diverse set of molecular determinants contributing to niche-specific adaptation and persistence of L. monocytogenes ST121 strains in food processing environments.

## INTRODUCTION

Listeria monocytogenes is a Gram-positive foodborne pathogen and the causative agent of listeriosis, a rare but severe disease associated with high mortality rates in humans. Especially immunocompromised and elderly individuals, infants, and pregnant women are susceptible to invasive listeriosis after consumption of contaminated food (1, 2).

L. monocytogenes is perfectly equipped to adapt to and survive in marine water, freshwater, sewage water, and soil or vegetation, as well as in food processing environments and food products (3). In niches as diverse as these, L. monocytogenes is able to tolerate and adapt to a variety of different stress conditions, including wide pH, salt concentration, and temperature ranges, low water activity, and different sanitizers: e.g., quaternary ammonium compounds, hydrogen peroxide, peracetic acid, and sodium hypochlorite (4, 5).

However, there is a high degree of strain divergence in stress response and environmental adaptation. The genome of L. monocytogenes is highly stable and conserved but sporadically interspersed by mobile chromosomal elements like prophages, transposons, or genomic islands (6–8). It has been shown that these mobile elements as well as plasmids comprise new genetic information, referred to as accessory gene content, and facilitate adaptation to new niches for certain L. monocytogenes strains. Interestingly, the accessory gene content is not evenly scattered across the chromosome but clustered in hypervariable hot spots. The functions of most of the genes within hypervariable hot spots remain to be determined (9–11).

One of these hypervariable hot spots in L. monocytogenes is located between the mutually conserved core genes *lmo0443* and *lmo0449*. Three different insertions between *lmo0443* and *lmo0449* have been identified in L. monocytogenes: the stress survival islet 1 (SSI-1) (12), a homologue of the *LMOf2365_0481* gene, and homologues of the Listeria innocua genes *lin0464* and *lin0465* (13). SSI-1 is an 8.7-kbp region consisting of the five genes *lmo0444*, *lmo0445*, *pva*, *gadD1*, and *gadT1*, which have been linked to tolerance toward acidic, salt, bile, and gastric stresses (12, 14–16). Furthermore, SSI-1 is important for growth in the food matrix (12). *LMOf2365_0481* is a 548-bp gene whose function is unknown. *lin0464* and *lin0465* are transcribed in the opposite direction and show 98% and 94% DNA identities to their homologues in L. innocua. The arrangement of *lin0464* and *lin0465* is conserved in L. innocua and L. monocytogenes (13). Lin0464 is a putative transcriptional regulator of the GntR family with a helix-turn-helix DNA-binding domain, and Lin0465 is predicted to be an intracellular PfpI protease of the DJ-1/PfpI protease superfamily with a type I glutamine amidotransferase-like domain characterized in Pyrococcus furiosus (17). Hein et al. showed that strains of sequence type 121 (ST121) harbor the *lin0464-lin0465* insert (13). This finding could be confirmed by two recent genome studies showing that the *lin0464-lin0465* insert is present in all analyzed ST121 strains (7, 18). L. monocytogenes strains of ST121 are often found to be abundant and to persist in food processing environments. This suggests that ST121 strains harbor specific genetic determinants supporting persistence and conferring adaptation to a categorical discriminative niche (13, 19–22).

We hypothesize that the *lin0464-lin0465* insert is a functional unit and contributes to specific stress response differently than SSI-1, potentially supporting the survival of L. monocytogenes in the food processing environment. First we investigated the prevalence of the different inserts (SSI-1, *lin0464-lin0465*, and *LMOf2365_0481*) in 476 L. monocytogenes strains isolated from humans, food products, and food processing environments and determined the sequence types of all strains harboring *lin0464-lin0465*. Additionally, we characterized all *lin0464-lin0465*-positive strains in the genome database GenBank and performed a phylogenetic analysis of the *lin0464-lin0465* insert. To elucidate the role of the intracellular PfpI protease Lin0465 in stress survival, we generated a *lin0465* deletion (Δ*lin0465*) mutant strain using the persistent L. monocytogenes strain 6179 (ST121, serotype 1/2a), which has repeatedly been isolated from an Irish cheese processing environment over a total period of 12 years (23). This strain was exposed to different stress conditions (acidic, gastric, cold, heat, osmotic, alkaline, oxidative, and antibiotic stresses). To test our hypothesis that *lin0464* and *lin0465* are a functional unit, we analyzed mRNA expression of *lin0465* in a strain devoid of the putative transcription factor *lin0464*.

## RESULTS

### Prevalence of SSI-1, *lin0464-lin0465*, and *LMOf2365_0481* among Listeria monocytogenes strains.

**FIG 1:**
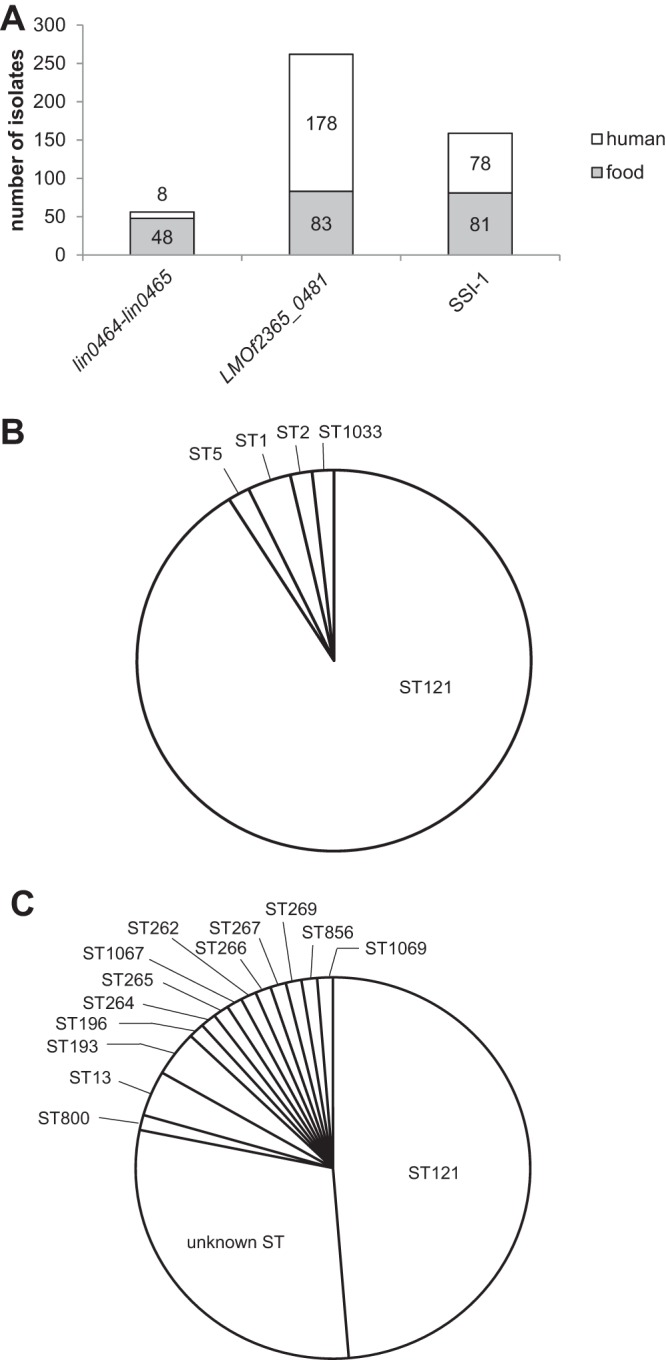
(A) Prevalence of *lin0464-lin0465*, *LMOf2365_0481*, and SSI-1 in L. monocytogenes strains (*n* = 476) isolated from humans, food, and a food processing environment (category food). (B) Sequence type (ST) distribution of L. monocytogenes strains harboring *lin0464-lin0465* (*n* = 56) detected in this study. (C) ST distribution in *lin0464-lin0465*-positive L. monocytogenes strains for which the genome is available in GenBank.

In total, 476 L. monocytogenes strains were screened for the presence of three different inserts in the genetically hypervariable region *lmo0443* to *lmo0449*: the *lin0464-lin0465* insert was harbored by 11.8% of strains (*n* = 56), the homologue of the *LMOf2365_0481* gene by 54.8% (*n* = 261), and stress survival islet 1 (SSI-1) by 33.3% (*n* = 159) (Fig. 1A; see Data Set S1 in the supplemental material).

The prevalence of the three inserts differs between human and food-related strains (including isolates from food and food processing environments). The human strains (*n* = 264) were isolated between 1959 and 2009 from 14 different countries and included 17 different patients' specimens. Only two strains were related to a listeriosis outbreak. The category of food-related strains (*n* = 212) included strains isolated between 1986 and 2016 from 29 countries and 74 isolation sites (13 for the food processing isolates and 61 for the food isolates [for more detail, see Data Set S1]). In human strains, the *lin0464-lin0465* insert was detected in 3.0% (*n* = 8), *LMOf2365_0481* in 67.4% (*n* = 178), and SSI-1 in 29.4% (*n* = 78). In food-related strains, *lin0464-lin0465* was present in 22.6% (*n* = 48), *LMOf2365_0481* in 39.2% (*n* = 83), and SSI-1 in 38.2% (*n* = 81). We did not observe any difference in the prevalences of the three different inserts between strains isolated from food and strains isolated from food processing environments.

Additionally, we detected 78 strains harboring the *lin0464-lin0465* insert, of which the genomes were available in GenBank, among them two strains (6179 and 4423) used in our study (see Data Set S2 in the supplemental material). The 78 strains included 20 food isolates, 17 strains isolated from food processing environments, and 4 human and 28 animal isolates. The source of nine strains was unknown. The nucleotide sequences of *lin0464* and *lin0465* of these 78 strains were highly similar to those of the homologous genes in strain 6179: 98.61 to 100% for *lin0464* and 96.56 to 100% for *lin0465*.

### The *lin0464-lin0465* insert is predominantly harbored by L. monocytogenes strains of ST121.

All L. monocytogenes strains harboring the *lin0464-lin0465* insert (*n* = 56) were subjected to multilocus sequence typing (MLST): 91.1% of the strains belong to ST121 (*n* = 51, clonal complex 121 [CC121], lineage II). The other *lin0464-lin0465*-positive strains are of lineage I: two strains of ST1 and one strain each of ST1033 (both CC1), ST2 (CC2) and ST5 (CC5) (Fig. 1B).

ST determination of the 78 strains of which the genome was available in GenBank revealed a slightly different result: 48.7% strains belong to ST121 (*n* = 38, CC121, lineage II), and 3.8% to ST13 (*n* = 3, CC13, lineage II) and ST193 (*n* = 3, CC193, lineage II). Additionally, we detected one strain each of the following STs: ST265, ST269, ST800, ST856, ST1067, and ST1069 (all lineage III), ST196 (CC193, lineage II), ST267 (CC267), and ST262 (CC262), ST264, and ST266 (all four not assigned to any lineages). However, the ST of 23 strains (29.1%), mainly animal isolates, was unknown (no hit in the ST database [Fig. 1C; Data Set S2]). Interestingly, all ST121 strains harbor the identical *lin0464* and *lin0465* genes (100% nucleotide identity), whereas the nucleotide identities of the homologous genes of the other strains are between 96.56 and 99.71%.

Analysis of the whole *lin0464-lin0465* insert of all 78 L. monocytogenes strains showed that the size of the insert varies between 1,949 and 1,952 bp, with a GC content of 36.46%, whereas the homologous insert in L. innocua (*n* = 4) is slightly shorter, harboring only 1,947 bp. The phylogenetic analysis showed that the *lin0464-lin0465* insert of the ST121 strains is identical and that the insert of the ST13 and CC193 strains has a higher similarity to that of the L. innocua strains than to those of the other strains (see Fig. S1 in the supplemental material).

### Growth and survival under stress conditions.

**FIG 2:**
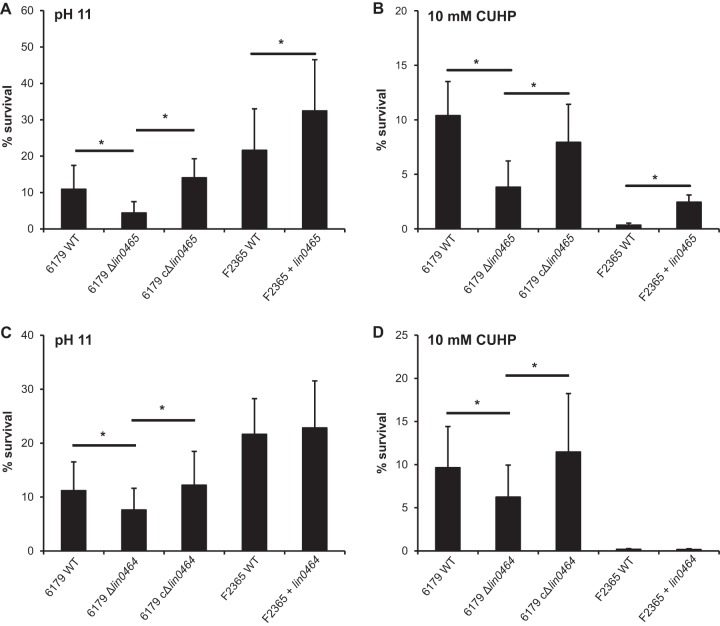
(A and B) Survival of the L. monocytogenes 6179 wild type (WT), 6179 Δ*lin0465* deletion mutant, complemented 6179 Δ*lin0465* deletion mutant (6179 cΔ*lin0465*), F2365 wild type, and F2365 expressing *lin0465* (F2365 + *lin0465*) under alkaline (pH 11) (A) and oxidative (10 mM CUHP) (B) stress conditions. (C and D) Survival of the L. monocytogenes 6179 wild type, 6179 Δ*lin0464* deletion mutant, 6179 cΔ*lin0464*, F2365 wild type, and F2365 expressing *lin0464* (F2365 + *lin0464*) under alkaline (pH 11) (C) and oxidative (10 mM CUHP) (D) stress conditions. Data are shown as a percentage of survival after exposure to stress for 2 h at 37°C. The percentage of survival was determined by CFU plate counting. Values represent mean values ± SD from five biological replicates performed in triplicate. *, *P* ≤ 0.05.

A significant difference between the wild-type and Δ*lin0465* deletion mutant strains was only observed in survival under alkaline and oxidative stress conditions (Fig. 2A and B). Deletion of *lin0465* had no effect on growth under cold, osmotic (see Fig. S2 in the supplemental material), antibiotic (see Table S2 in the supplemental material), and benzalkonium chloride (data not shown) stress conditions and on the survival under acidic, gastric, or heat stress (see Table S1 in the supplemental material) but decreased significantly the survival of L. monocytogenes under alkaline and oxidative stresses. This phenotype could be reversed in the complemented Δ*lin0465* deletion mutant (cΔ*lin0465*) strain (by introducing *lin0465* on a constitutive expression vector [Fig. 2A and B]).

Additionally, we introduced *lin0465* into L. monocytogenes strain F2365, which harbors the *LMOf2365_0481* insert. The overall survival of the F2365 wild-type strain was higher under alkaline stress conditions but lower under oxidative stress compared to the 6179 wild-type strain. Constitutive expression of *lin0465* significantly increased the survival of strain F2365 under both alkaline and oxidative stress conditions (Fig. 2A and B).

In the next step, we tested the role of the putative transcription factor Lin0464 in alkaline and oxidative stress responses. In parallel to the case for Lin0465, we detected significantly decreased survival of the Δ*lin0464* deletion mutant compared to the wild-type strain and increased survival of the complemented strain compared to the deletion mutant strain (Fig. 2C and D). However, the effect of the deletion of *lin0465* on the survival of strain 6179 under alkaline and oxidative stress conditions was higher than that of the deletion of *lin0464*.

As expected, the constitutive expression of *lin0464* in strain F2365 had no effect on the survival rate under alkaline and oxidative stress conditions, indicating that the expression of the transcription factor *lin0464* alone has no influence on survival (Fig. 2C and D).

These results show that *lin0464* and *lin0465* support survival of L. monocytogenes under alkaline and oxidative stresses and suggest that *lin0464* and *lin0465* are a functional unit.

### Expression of *lin0464* and *lin0465* in response to oxidative stress.

**FIG 3:**
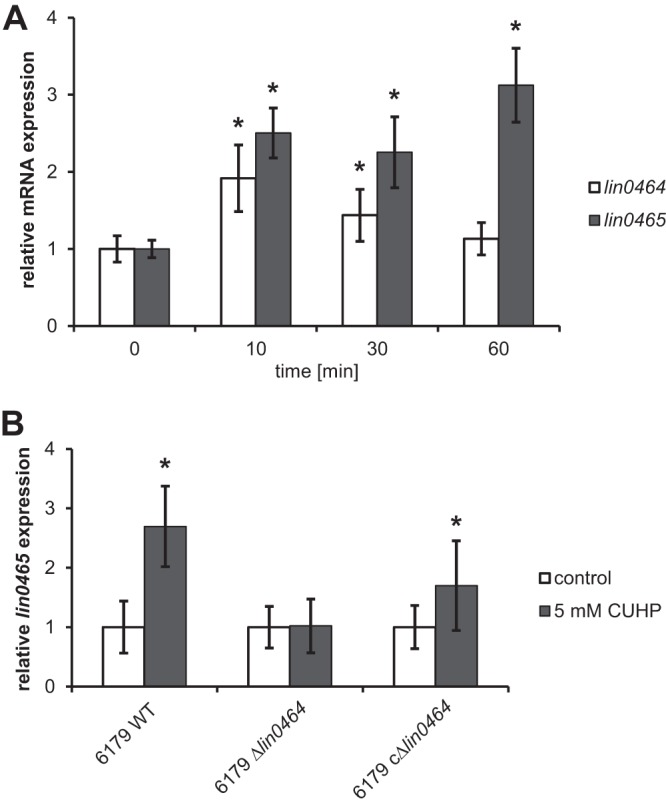
(A) mRNA expression of *lin0464* and *lin0465* in the L. monocytogenes 6179 wild-type (WT) strain incubated without (0 min [control]) and with 5 mM CUHP for 10, 30, and 60 min at 20°C. (B) mRNA expression of the putative protease gene *lin0465* in the L. monocytogenes 6179 wild type, 6179 Δ*lin0464* deletion mutant, and complemented 6179 Δ*lin0464* deletion mutant (6179 cΔ*lin0464*) incubated without (control) and with 5 mM CUHP for 10 min at 20°C. Values were normalized to 16S rRNA gene expression levels and are presented as *x*-fold of the control. Data represent mean values ± SD from two biological replicates performed and measured in duplicate. *, *P* ≤ 0.05 versus control.

The expression of both genes (*lin0464* and *lin0465*) was already significantly upregulated after 10 min of exposure to oxidative stress, suggesting that the *lin0464-lin0465* insert contributes to a rapid stress response. While transcription of *lin0464* decreases over time, transcription of *lin0465* continues to increase up to 3-fold after 60 min (Fig. 3A).

To test our hypothesis that the putative transcription factor Lin0464 regulates the transcription of Lin0465, we used a strain devoid of *lin0464*. Transcription of *lin0465* after 10 min of exposure to oxidative stress was significantly different in the wild-type and the Δ*lin0464* deletion mutant strains (Fig. 3B). The expression of *lin0465* was not induced under oxidative stress conditions in the Δ*lin0464* deletion mutant strain but again was induced in the complemented Δ*lin0464* deletion mutant (cΔ*lin0464*) strain. These data strongly support our hypothesis that the transcription factor gene *lin0464* and the putative protease gene *lin0465* are a functional unit. We propose to name this genomic islet stress survival islet 2 (SSI-2).

As expected, transcription of *lin0464* was undetectable in the 6179 Δ*lin0464* deletion mutant strain and increased in the complemented strain (6179 cΔ*lin0464*) (700-fold [see Table S3 in the supplemental material]). However, the expression of *lin0465* in the complemented Δ*lin0464* deletion mutant strain was comparable to the expression in the 6179 wild-type strain with and without oxidative stress.

### The expression of *lin0464* and *lin0465* is independently regulated from σ^B^.

**FIG 4:**
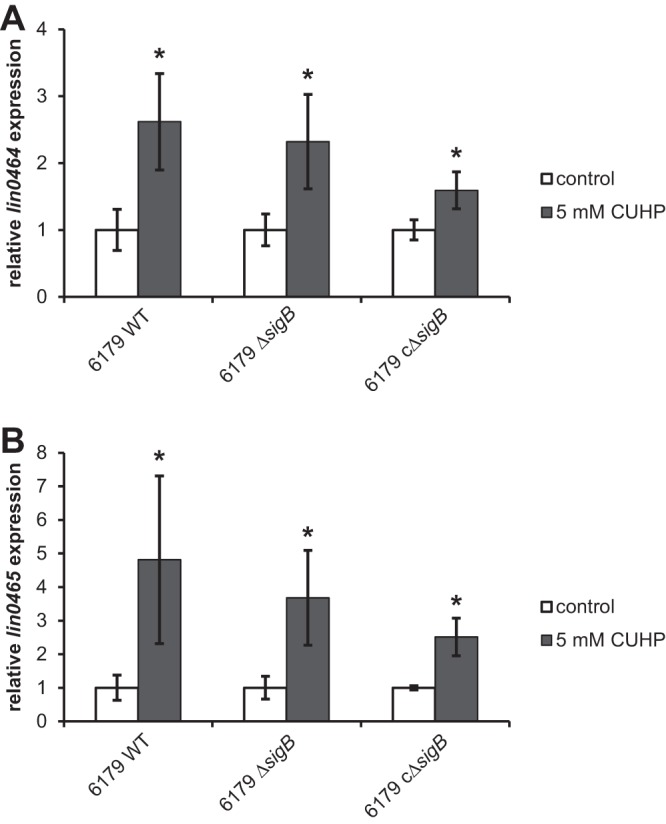
mRNA expression of *lin0464* (A) and *lin0465* (B) in the L. monocytogenes 6179 wild type (WT), *sigB* deletion mutant (6179 Δ*sigB*), and complemented 6179 *sigB* deletion mutant (6179 cΔ*sigB*) incubated without (control) and with 5 mM CUHP for 10 min at 20°C. Values were normalized to 16S rRNA gene expression levels and are presented as *x*-fold of the control. Data represent mean values ± SD from two biological replicates performed and measured in duplicate. *, *P* ≤ 0.05 versus control.

We additionally addressed the question of whether *lin0464* and *lin0465* are regulated by the general transcriptional response mechanism targeting genes involved in stress survival under the control of the alternative sigma factor σ^B^. The loss of σ^B^ did not affect transcription of *lin0464* and *lin0465* since we could observe a significant upregulation of both genes after exposure to oxidative stress in the *sigB* deletion mutant (Δ*sigB*) strain (Fig. 4A and B; Table S3).

## DISCUSSION

In this study, we characterized the novel stress survival islet SSI-2 in L. monocytogenes. SSI-2 consists of two genes—the transcription factor gene *lin0464* and the PfpI protease gene *lin0465*—and is present in the hypervariable genetic hot spot *lmo0443* to *lmo0449*, which harbors three inserts in total: SSI-1, SSI-2, and a homologue of the gene *LMOf2365_0481*.

Our prevalence study including 476 strains gives indications that SSI-2 is involved in a different stress response than SSI-1. SSI-1, supporting survival under salt, acidic, bile, and gastric stress conditions (12), is equally distributed among human clinical isolates and strains isolated from food and food processing environments, whereas SSI-2 is predominantly present in L. monocytogenes strains isolated from food and food processing environments (84%). The determination of the STs of all SSI-2-positive L. monocytogenes strains (from the PCR screening and genome database analysis) further strengthens our hypothesis. SSI-2 is mainly harbored by strains of ST121 (CC121, lineage II), whereas SSI-1 is known to be present in strains of diverse STs and lineages (19, 24). Strains of CC121 are highly abundant and among the most prevalent clones isolated from food or food processing environments, but they are underrepresented among clinical isolates due to an internalin A gene truncation leading to attenuated virulence (19). In two recent genome studies including 70 ST121 strains in total, we showed that SSI-2 is present in all ST121 strains. Moreover, SSI-2 is highly conserved in ST121 strains sharing 100% nucleotide identity (7, 18). We could confirm these findings in the present study.

SSI-2 also seems to be occasionally present in L. monocytogenes strains of other STs, including one new ST (ST1033): however, with a lower nucleotide identity (97 to 99%) compared to SSI-2 of ST121 strains. This is in line with the study of Ebner et al. reporting a few non-ST121 strains harboring SSI-2 (24). The ST of a surprisingly large number of SSI-2-positive strains (*n* = 23) identified by the genome database analysis is unknown. Most of these strains are animal isolates derived from one genome study. Since we cannot exclude that the quality of the genome sequence influenced the ST determination, the ST of these strains has to be characterized by classical multilocus sequence typing (MLST) PCR. Besides strains of lineage II, we also detected SSI-2-positive strains belonging to lineage I (*n* = 4; three human strains and one from a food processing environment) and to lineage III (*n* = 6; five animal isolates and one food isolate). Our findings are consistent with previous studies reporting diverse distribution of lineage I strains among human, food, and food-related isolates and an increased incidence of lineage III strains among animal isolates (25, 26). In conclusion, we can state that SSI-2 is predominantly but not exclusively present in strains of ST121.

Genomic islets such as SSI-2 are part of the accessory genome, confer quick improved adaptation to environmental variations, and are consistently interchanged by horizontal gene transfer, habitually including remnants of mobility-enabling sequences (27).

*lin0464* and *lin0465* in L. monocytogenes are homologues to the genes in L. innocua, with 98% and 94% DNA identity, respectively. There is growing evidence that all L. innocua strains habor SSI-2 (28). The whole SSI-2 of L. innocua is slightly shorter; however the lengths of *lin0464* and *lin0465* are identical. Our phylogenetic analysis revealed the highest similarity of L. innocua SSI-2 to those of the ST13 and CC193 strains (lineage II, all food isolates).

Given that L. monocytogenes and L. innocua are more closely related to each other than to other Listeria species and coexist in the same ecological niches, SSI-2 was most plausibly integrated into the L. monocytogenes genome in an elemental horizontal gene transfer event from L. innocua (29). The fact that the GC content of SSI-2 (36.46%) is more similar to that of L. innocua (37.4% on average) than that of L. monocytogenes (38% on average) supports the hypothesis of horizontal gene transfer. However, we could not identify obvious mobility factors such as integrases, transposases, or insertion sequence elements in SSI-2. This is consistent with the findings of Ryan et al., who reported the absence of mobility factors also in SSI-1 (12).

Unlike SSI-1 and SSI-2, we mainly detected the single-gene insert *LMOf2365_0481* in human L. monocytogenes isolates. *LMOf2365_0481* harbors a domain of unknown function, which is often associated with a WGR domain (PF05406), a putative nucleic acid-binding domain found in various transcriptional regulators: e.g., MolR (YehH) in Escherichia coli (30). However, the role of *LMOf2365_0481* in stress response, niche adaptation, and virulence still remains to be elucidated and is the focus of our ongoing research.

The phenotypical characterization of *lin0464* and *lin0465* proved our hypothesis that SSI-2 is involved in stress response differently than SSI-1. We showed that the protease Lin0465 and, to a lesser extent, the transcription factor Lin0464 only support survival under alkaline and oxidative stress conditions and are not like SSI-1 involved in salt, acidic, and gastric stress responses. Of note, SSI-2 seems not to be involved in tolerance to quaternary ammonium compounds.

L. monocytogenes faces alkaline and oxidative stresses during cleaning and sanitation procedures in the food processing environment (31–33). Oxidizing agents such as hydrogen peroxide, chlorine dioxide, peracetic acid, and sodium hypochlorite are frequently applied as antimicrobials. These low-molecular-weight compounds can easily pass cell membranes and cause oxidative stress by exerting their basic mechanism of action, the oxidation of cellular components (4, 32). In order to prevent oxidative damage caused to macromolecules leading to increased rates of mutagenesis and consequently to cell death, nonenzymatic and enzymatic protection as well as repair and detoxification mechanisms are essential (34). Nonenzymatic protection also involves proteases, which in general have an important function in orchestrating cellular reactions during stress response toward host-associated and non-host-associated stress conditions by degrading misfolded proteins, preventing accumulation of potentially toxic proteins and regulating chaperone and stress-related protein levels. Several proteases have successfully been identified to have a major role in stress response in L. monocytogenes, like the serine protease HtrA, essential for survival under heat, acid, and penicillin stress conditions (28), or the ClpP serine protease, known to be involved in heat and osmotic stress responses (35, 36).

Homologous proteins of the PfpI protease Lin0465 have already been linked to stress response in other bacterial species: e.g., in Pseudomonas aeruginosa, the protease PfpI is involved in antibiotic, UV, osmotic, and thermal stress responses (37, 38), and in E. coli, the PfpI protease YhbO protects the bacteria against high temperature, extreme pH, and UV irradiation (39). Searching L. monocytogenes genome data for the presence of proteins harboring a DJ-1/PfpI domain revealed that the L. monocytogenes genome encodes an additional PfpI protease, Lmo2256, with 20% amino acid identity to Lin0465. Transcriptome sequencing showed that expression of *lmo2256* is increased under salt stress and long-term survival in L. monocytogenes, suggesting also a role of this PfpI protease in stress response (40, 41). However, the function of Lmo2256 is unknown.

In this study, we show an additional role of the PfpI protease in bacteria as being involved in alkaline and oxidative stress responses in L. monocytogenes. Therefore, SSI-2-positive strains might have an advantage in surviving in the food processing environment. This is in line with the fact that ST121 strains are highly abundant in the food processing environment and subsequently in food.

Our study provides several indications that *lin0464* and *lin0465* are a functional unit, justifying the term “stress survival islet”: (i) both genes support survival under the same stress conditions, (ii) mRNA expression of both genes is increased under oxidative stress, (iii) the time frame of increased transcription of the putative protease gene *lin0465* due to oxidative stress is longer than that of *lin0464*, (iii) the constitutive expression of the transcription factor *lin0464* alone in a strain devoid of SSI-2 did not alter the survival rate under alkaline and oxidative stresses (in contrast to *lin0465*), and (iv) finally we show that mRNA expression of *lin0465* under stress conditions is regulated by Lin0464, confirming its function as a positive gene regulator.

Also SSI-1 includes a transcription factor gene, *lmo0445*, which regulates the expression of the islet genes *lmo0444*, *lmo0446*, *lmo0447*, and *lmo0448* (12). However, in contrast to our study, Ryan et al. already observed regulation of the islet genes under nonstress conditions (in the stationary and exponential growth phases) (12). In our study, the basal transcription of *lin0465* was not altered in the *lin0464* mutant strain. Even in the strain constitutively expressing the transcription factor gene *lin0464*, the transcription of the protease gene *lin0465* was only increased under stress conditions. This indicates that other transcriptional factors might be involved in the gene regulation of *lin0465* under nonstress conditions.

Additionally, we detected that regulation of SSI-2 is independent from the alternative stress sigma factor σ^B^. σ^B^ is essential for the survival of L. monocytogenes under stress conditions encountered in non-host-associated environments by mediating transcriptional initiation of stress-related genes, including all genes of SSI-1 (12, 42–44). Besides σ^B^, there are additional alternative sigma factors, such as σ^H^ or σ^L^, which could be involved in SSI-2 regulation. The alternative sigma factor σ^H^ encodes a potentially pH-regulated transcriptional regulator that plays a role in survival under nutrient limitation and alkaline stresses, whereas σ^L^ is required for efficient growth at low temperatures and in the presence of various stresses such as organic acids, antibiotics, and toxins (45–48). The involvement of σ^H^ or σ^L^ in the regulation of the genes of SSI-2 remains to be elucidated. At the moment, we can only state that SSI-2 is, as are many genomic islets and islands (49), a self-regulating islet independent from σ^B^ transcription.

In conclusion, we identified a novel stress survival islet, SSI-2, in L. monocytogenes, which consists of the two genes *lin0464* and *lin0465*, forming an internally regulated and functional unit that supports survival under alkaline and oxidative conditions. SSI-2 is highly conserved and predominantly found in L. monocytogenes strains of ST121. Strains of ST121 are among the most abundant L. monocytogenes isolates in the food processing environment and food. Moreover, some of them have been reported to be persistent, since genetically indistinguishable ST121 strains have been repeatedly isolated from food production plants over extended time periods (50, 51). The genome of ST121 strains is highly conserved and harbors other genetic features besides SSI-2 that support survival in the food processing environment, like plasmids and the novel transposon Tn*6188*, conferring tolerance toward quaternary ammonium compounds (7, 9, 52). Collectively, these specific properties of ST121 strains facilitate survival and promote adaptation to encountered niches and can potentially lead to persistence in food processing environments. Understanding the genetic features of persistence is of great importance in order to control the occurrence of L. monocytogenes in food processing plants, to limit food contamination, and to subsequently avoid listeriosis.

## MATERIALS AND METHODS

### Bacterial strains.

The L. monocytogenes strains used in the prevalence study (*n* = 476) are shown in Data Set S1. The selected strain set consisted of human strains (*n* = 265) and strains from food and food processing environments (category food, *n* = 212), all available at the Institute for Milk Hygiene at the University of Veterinary Medicine Vienna.

Strain 6179, a cheese isolate from Ireland of serotype 1/2a and multilocus sequence type 121 (ST121) (21, 51) harboring insert *lin0464-lin0465* (7), and F2365, a cheese isolate from California of serotype 4b and ST1 (53) harboring insert *LMOf2365_0481*, were used as positive controls for the *lmo0443-lmo0449* screening PCR and for the generation of deletion mutants and complemented strains. Additionally, EGD-e (ATCC BAA-679), a rabbit isolate of serotype 1/2a and ST35 harboring insert SSI-1, was used as a positive control for the *lmo0443-lmo0449* screening PCR.

### DNA isolation.

All strains were stored at −80°C in brain heart infusion supplemented with yeast (BHI-Y) and 60% glycerol. Stock cultures were streaked out onto tryptic soy agar (TSA) and incubated at 37°C overnight. DNA was isolated from 1 ml of overnight culture (in BHI-Y at 37°C with shaking at 125 rpm) using the NucleoSpin tissue kit.

### Screening PCR.

In total, 476 human, food, and food-associated L. monocytogenes strains were screened for the presence of inserts in the intergenic region between *lmo0443* and *lmo0449*. PCR primers targeting the flanking genes *lmo0443* and *lmo0449* were used according to Hein et al. (13) (Table 1). Due to the different sizes of the inserts, two different PCR approaches were performed. For the detection of *lin0464-lin0465* and *LMOf2365_0481*, PCR was performed using Platinum *Taq* DNA polymerase. The PCR conditions were as follows: 0.2 μM each primer, 2 mM MgCl_2_, 1 mM deoxynucleoside triphosphates (dNTPs), 1 U Platinum *Taq* DNA polymerase, 10× PCR buffer, diethyl pyrocarbonate (DEPC)-treated water, and 1 μl DNA template in a final volume of 25 μl. The PCR cycling conditions were as follows: initial denaturation at 94°C for 5 min, followed by 30 cycles of denaturation at 94°C for 30 s, annealing at 55°C for 30 s, and elongation at 72°C for 2 min, followed by final elongation for 5 min at 72°C and hold at 4°C. For the detection of SSI-1 (8.7 kbp), a long-range PCR using Long PCR enzyme mix was conducted with 1 mM dNTPs, 1 μM each primer, 10× PCR buffer (with 15 mM MgCl_2_), 2.5 U Long PCR enzyme mix, DEPC-treated water, and 2 μl DNA template in a final volume of 25 μl. The PCR cycling conditions were as follows: 3 min at 94°C (initial denaturation), followed by 10 cycles of denaturation at 94°C for 20 s, annealing at 55°C for 30 s, and elongation at 68°C for 7 min, followed by 25 cycles of denaturation at 94°C for 20 s, annealing at 55°C for 30 s, and elongation at 68°C for 12 min, with final elongation at 68°C for 10 min and hold at 4°C. Negative controls (DEPC-treated water) and positive controls (genomic DNA from L. monocytogenes strains 6179, F2365, and EGDe) were included in all PCRs. The presence and size of inserts were analyzed by agarose gel electrophoresis and visualized using peqGREEN staining.

**TABLE 1:** Primers used for screening PCR, qRT-PCR, and the generation of deletion mutant and complemented strains

Gene target	Method	Primer	Sequence (5′→3′)
*lmo0443*^*a*^	Screening PCR	Forward	GGCACAATGAGCGAATTG
*lmo0449*^*a*^	Screening PCR	Reverse	GTCCTTCTGGAACATTGC
16S rRNA gene	qRT-PCR	Forward	TTAGCTAGTTGGTAGGGTAATGGC
		Reverse	CAGTACTTTACGATCCGAAAACCT
*lin0464*	qRT-PCR	Forward	CAGCAGACCTTTCCGCTATC
		Reverse	TGGTTCGCTTTTAGCTCGAT
*lin0465*	qRT-PCR	Forward	GATGACGATAGTGCCCGATT
		Reverse	GCGAGAAGCTCACTTGCTTT
*sigB*	qRT-PCR	Forward	GCGCCGAATCAAAGAGTTAG
		Reverse	TTTCCCATTTCCATTGCTTC
*lin0464*	Generation of deletion mutant	SoeA	ATGGAATTCTATCGTCATCCCACCCA
		SoeB	ATAATAGCCACCTTTCAAAATTAC
		SoeC	GTAATTTTGAAAGGTGGCTATTATTGCTTGTTTTCATCAAAAAAACACG
		SoeD	AGGCTGCAGTGATGTTGGTGATGTTG
		SoeE	CTTCTGCATCTTTACGGAAGCG
		SoeZ	CGCTTCCGTAAAGATGCAGAAG
*lin0465*	Generation of deletion mutant	SoeA	ATGGAATTCGGAGTTGAACGAATA
		SoeB	ATATTTCCTCCATTTTCTATTAAT
		SoeC	ATTAATAGAAAATGGAGGAAATATTTAAGTGAGGCGGCTAATCTACAA
		SoeD	AGGCTGCAGAGATGGCGCTGTAATT
		SoeE	GTGCTATTCTTACCATCCAT
		SoeZ	ATAAGGATAGCGGAAAGGTC
*sigB*	Generation of deletion mutant	SoeA	ATGGAATTCACTAATTCCGTAAAGC
		SoeB	AATGAAAAGCAGGTGGAGGAGA
		SoeC	AATGAAAAGCAGGTGGAGGAGAATGAACAAGGCAGTTGAATC
		SoeD	AGGCTGCAGGTGTCTTCGTTAAGTAATC
		SoeE	CGATTGCAACCGATATTTCTG
		SoeZ	CACAGGATTGTCAGATATCATC
*lin0464*	Complementation	Forward	CGGCCATGGAAGTTGATAGGCTTAT
		Reverse	CGGTCTAGACGTTTAGATGATATGGC
*lin0465*	Complementation	Forward	CGGCCATGGTTACAATTTATGTTTAC
		Reverse	CGGTCTAGAGCAAGCGACCAACCTACTTTG
*sigB*	Complementation	Forward	CGGCCATGGCAAAAGTATCTCAACC
		Reverse	CGGCTGCAGCATCCCCGCAGTATTG

a Primers according to Hein et al. (13).

### MLST.

For all L. monocytogenes strains harboring the *lin0464-lin0465* insert, multilocus sequence typing (MLST) was performed based on seven housekeeping genes: *abcZ* (ABC transporter), *bglA* (β-glucosidase), *cat* (catalase), *dapE* (succinyl diaminopimelate desuccinylase), *dat* (d-amino acid aminotransferase), *ldh* (l-lactate dehydrogenase), and *lhkA* (histidine kinase).

PCR was performed as suggested on the L. monocytogenes MLST database (http://bigsdb.pasteur.fr/listeria/primers_used.html). The PCR cycling conditions were as follows: initial denaturation at 94°C for 4 min, followed by 35 cycles of denaturation at 94°C for 30 s, annealing at 52°C (for all genes except *bglA* [45°C]) for 30 s, and elongation at 72°C for 1 min, with final elongation at 72°C for 10 min. PCR products were purified and sequenced (LGC Genomics, Berlin, Germany), and ST allele profiles and classification of the obtained STs into clonal complexes were determined using the L. monocytogenes MLST database. The new ST allele combination was sent for validation to the Listeria MLST database.

Additional strains harboring the *lin0464-lin0465* insert (*n* = 78) were retrieved from GenBank by BLASTN using the NCBI genomes and whole-genome shotgun contigs (WGS) database (54). Determination of the ST of these strains was performed with the MLST tool available on the Center for Genomic Epidemiology website (https://cge.cbs.dtu.dk/services/MLST/) (55). For each strain, the SSI-2 nucleotide sequence was retrieved using BLASTN and aligned using MUSCLE implemented in MEGA7 (56). Additionally we included the SSI-2 sequences of four L. innocua strains (ATCC 33091, 12KSM, MOD1_LS888, and 9KSM). The maximum likelihood method based on the Tamura-Nei evolutionary model was used for the molecular phylogenetic analysis (57).

### Generation of Δ*lin0464*, Δ*lin0465*, and Δ*sigB* deletion mutants and complemented strains.

In order to generate Δ*lin0464*, Δ*lin0465*, and Δ*sigB* nonpolar deletion mutants in the L. monocytogenes 6179 wild type, the splicing by overlap extension PCR technique (SOEing-PCR) and the temperature-sensitive shuttle vector pKSV7 (58), conferring chloramphenicol resistance, were used according to Rychli et al. (59) (Table 2). Primers were designed (SoeA to -D) to amplify two DNA segments (SoeAB and SoeCD) flanking the genomic region targeted for deletion and for the screening PCR (Table 1). PCRs were performed using Phusion Green high-fidelity DNA polymerase.

**TABLE 2:** L. monocytogenes strains and plasmids used in this study

*S*train or plasmid	Relevant characteristic(s)	Source or reference
Strains		
6179	Wild type, ST121	21, 51
F2365	Wild type, ST1	53
6179 Δ*lin0464*	*lin0464* deletion mutant	This study
6179 cΔ*lin0464*	Complemented Δ*lin0464* mutant strain	This study
6179 Δ*lin0465*	*lin0465* deletion mutant	This study
6179 cΔ*lin0465*	Complemented Δ*lin0465* mutant strain	This study
6179 Δ*sigB*	*sigB* deletion mutant	This study
6179 cΔ*sigB*	Complemented Δ*sigB* mutant strain	This study
F2365 *lin0464*	F2365 constitutively expressing *lin0464*	This study
F2365 *lin0465*	F2365 constitutively expressing *lin0465*	This study
Plasmids		
pKSV7	Chloramphenicol resistant, temp sensitive	58
pNZ44	Vector leading to constitutive expression of target gene, chloramphenicol resistant	60

For the complementation of the *lin0464*, *lin0465*, and *sigB* mutant strains, we used the vector pNZ44 (60), leading to constitutive gene expression (60). DNA was amplified from the L. monocytogenes 6179 wild type using Phusion Green high-fidelity DNA polymerase and specific primer pairs (Table 1). The vector pNZ44 and the *lin0464*, *lin0465*, and *sigB* PCR products were digested with two restriction enzymes, respectively (*lin0464* and *lin0465*, XbaI and NcoI; *sigB*, NcoI and PstI), ligated using T4 DNA ligase, and transformed into competent Escherichia coli (StrataClone SoloPack; Agilent Technologies). The plasmid containing the gene of interest was then electroporated into competent 6179 Δ*lin0464*, Δ*lin0465*, and Δ*sigB* deletion mutant strains and the F2365 wild-type strain. Positive transformants were selected on TSA supplemented with 10 μg/ml chloramphenicol and confirmed by PCR.

### Growth under stress conditions.

The L. monocytogenes 6179 wild type and 6179 Δ*lin0465* deletion mutant strain were grown in BHI-Y overnight at 37°C. The optical density at 600 nm (OD_600_) of 0.2 was adjusted, and the bacterial cells were grown in either BHI-Y or defined minimal medium [DMM: RPMI 1640 supplemented with 0.088 g/liter ferric(III)citrate] at 10°C (cold stress), 37°C, and 44°C (heat stress) or BHI-Y supplemented with 5% NaCl at 10 and 37°C (osmotic stress). OD_600_ was measured every hour for a minimum of 24 h.

The MIC determination of benzalkonium chloride was performed according to Müller et al. (52) using benzalkonium chloride concentrations of 0, 2, 5, 10, 15, 20, 25, and 30 mg/liter.

### Survival under stress conditions.

The L. monocytogenes 6179 wild type and 6179 Δ*lin0465* deletion mutant strain were grown to the stationary growth phase in 8 ml BHI-Y and adjusted to an OD_600_ of 0.1 in a total volume of 10 ml DMM adjusted to either pH 2.5 (with 1 M HCl) or pH 11 (with 2.5 M NaOH) or containing 10 mM cumene hydroperoxide (CUHP) and incubated for 2 h at 37°C. Furthermore, bacteria were incubated for 2 h at 37°C in synthetic gastric fluid according to Cotter et al. (61). Bacteria incubated only in DMM were used as a control. Heat stress was applied by incubating bacteria for 10 and 30 min in DMM at 55°C. CFU were determined by counting after serial plating on TSA plates in triplicates. Each experiment was performed three times. Percentages of survival were calculated by dividing the counted, untransformed CFU of cultures exposed to stress by the counted, untransformed CFU of control cultures not exposed to stress.

Additionally, we determined survival under alkaline and oxidative conditions using the 6179 wild type, 6179 Δ*lin0465* deletion mutant and complemented strain (6179 cΔ*lin0465*), 6179 Δ*lin0464* deletion mutant and complemented strain (6179 cΔ*lin0464*), F2365 wild type, and F2365 *lin0464* and F2365 *lin0465*. These experiments were repeated five times.

### Antibiotic susceptibility testing.

Antibiotic susceptibility of the L. monocytogenes 6179 wild type and 6179 Δ*lin0465* deletion mutant strain was determined by disk diffusion test on Mueller-Hinton agar supplemented with 2.5% sheep blood incubated at 37°C for 24 h (Table S2). The results were interpreted according to EUCAST (62).

### Isolation of mRNA.

A single colony of the specific L. monocytogenes strains was grown in 8 ml BHI at 37°C with shaking (125 rpm) for 8 h. Bacterial cultures were adjusted to an OD_600_ of 0.1 in a final volume of 35 ml of DMM and incubated at 20°C for 17 h. Cells were exposed to oxidative stress (5 mM CUHP in 15 ml DMM) for 10, 30, and 60 min (for the 6179 wild type) and 10 min for the 6179 Δ*lin0464* deletion mutant and complemented strain and 6179 Δ*sigB* deletion mutant and complemented strain at 20°C, harvested by centrifugation (3,220 × *g*, 10 min, 20°C), resuspended in 350 μl RNAlater solution, and stored at 4°C until RNA isolation. RNA isolation was performed according to Rychli et al. (63). A PCR targeting the 16S rRNA gene was performed using BiomixRed to confirm the absence of DNA. The PCR cycling conditions were as follows: initial denaturation at 95°C for 5 min, followed by 30 cycles of denaturation at 95°C for 15 s, annealing at 60°C for 30 s, and elongation at 72°C for 30 s, with final elongation at 72°C for 2 min and hold at 4°C. Primers are listed in Table 1. RNA amounts between 25 and 300 ng were used for cDNA synthesis using the RevertAid H Minus first-strand cDNA synthesis kit according to the manufacturer's protocol.

### qRT-PCR.

Primers targeting the L. monocytogenes 16S rRNA, *lin0464*, and *lin0465* genes were designed using Primer3 (v.0.4.0) (Table 1). For quantitative reverse transcription-PCR (qRT-PCR), the Brilliant III Ultra Fast SYBR green qPCR master mix with Low ROX and the Stratagene Mx3000P cycler was used. The cycling conditions were as follows: initial denaturation at 95°C for 3 min, followed by 40 cycles of denaturation at 95°C for 15 s, annealing at 60°C for 20 s, and elongation at 72°C for 20 s. Subsequently, a dissociation curve was established (55 to 95°C, 0.1°C/s). As an internal amplification control and for calculation of the respective primer efficiencies, a dilution series of genomic 6179 wild-type DNA (1 to 10^−6^ ng/μl) was used. Data were analyzed using Mx3000P MxPro software (Stratagene). Each sample was measured in duplicates, and relative quantification was performed using the comparative threshold cycle (*C_T_*) method. Values, given as *x*-fold of the 6179 wild-type control were normalized to 16S rRNA gene as an internal reference. Mean values ± standard deviations (SD) from three biological replicates performed in duplicate and measured in duplicate were calculated.

### Statistical analysis.

Microsoft Excel 2007 and SPSS.20 software were used for statistical analysis. The Brown-Forsythe and Welch tests were used to confirm the variance homogeneity. *t* tests with independent variables were used to compare the survival or gene expression between two groups, and a *post hoc* test (Tukey's honestly significant difference [HSD] in the case of variance homogeneity and Games-Howell in the case of variance inhomogeneity) was used to determine significant differences between the survival of more than two strains. *P* values of ≤0.05 were considered to be statistically significant.

## Supplementary Material

Supplemental material
