# Supplementary material for: Stress Survival Islet 2, Predominantly Present in Listeria monocytogenes Strains of Sequence Type 121, Is Involved in the Alkaline and Oxidative Stress Responses
Source: Appl Environ Microbiol. 2017 Aug 1;83(16):e00827-17. doi: 10.1128/AEM.00827-17 (PMC5541211; doi:10.1128/AEM.00827-17)
Supplement: Supplemental material [file supp_83_16_e00827-17__index.html]

Stress Survival Islet 2, Predominantly Present in Listeria monocytogenes Strains of Sequence Type 121, Is Involved in the Alkaline and Oxidative Stress Responses — Supplemental material 

# Stress Survival Islet 2, Predominantly Present in Listeria monocytogenes Strains of Sequence Type 121, Is Involved in the Alkaline and Oxidative Stress Responses

## Supplemental material

- Supplemental file 1 -

  Molecular phylogenetic analysis of SSI-2 by the maximum-likelihood method based on the Tamura-Nei model (Fig. S1); growth curves for *L. monocytogenes* 6179 wild-type and *lin0465* deletion mutant strain in BHI-Y and in DMM at 10°C, 37°C, and 44°C and in BHI-Y supplemented with 5% NaCl at 10°C and 37°C (Fig. S2); survival of *L. monocytogenes* 6179 wild-type and *lin0465* deletion mutant strains under acidic, gastric, and heat stress conditions (Table S1); antibiotic susceptibilities of 6179 wild-type and *lin0465* deletion mutant strains (Table S2); expression of *lin0464* and *sigB* genes in *L. monocytogenes* 6179 wild-type, deletion mutant, and complemented deletion mutant strains (Table S3).

  PDF, 1.2M
- Supplemental file 2 -

  Prevalence of the three different inserts (SSI-1, *lin0464*, and *lin0465*) and the *LMOf2365\_0481* gene in the hypervariable genetic hot spot *lmo0443*–*lmo0449* in 477 *L. monocytogenes* strains as determined by PCR (Data Set S1) .

  XLSX, 40K
- Supplemental file 3 -

  Sequence types of *lin0464*-*lin0465*-positive *L. monocytogenes* strains retrieved from GenBank by BlastN search, and nucleotide sequence identity and sequence query of *lin0464* and *lin0465* versus the homologous genes of strain 6179 (Data Set S2).

  XLSX, 19K
