# Supplementary material for: Stress Survival Islet 2, Predominantly Present in Listeria monocytogenes Strains of Sequence Type 121, Is Involved in the Alkaline and Oxidative Stress Responses
Source: Appl Environ Microbiol. 2017 Aug 1;83(16):e00827-17. doi: 10.1128/AEM.00827-17 (PMC5541211; doi:10.1128/AEM.00827-17)
Supplement: Supplemental material [file AEM.00827-17_zam999117995s1.pdf]

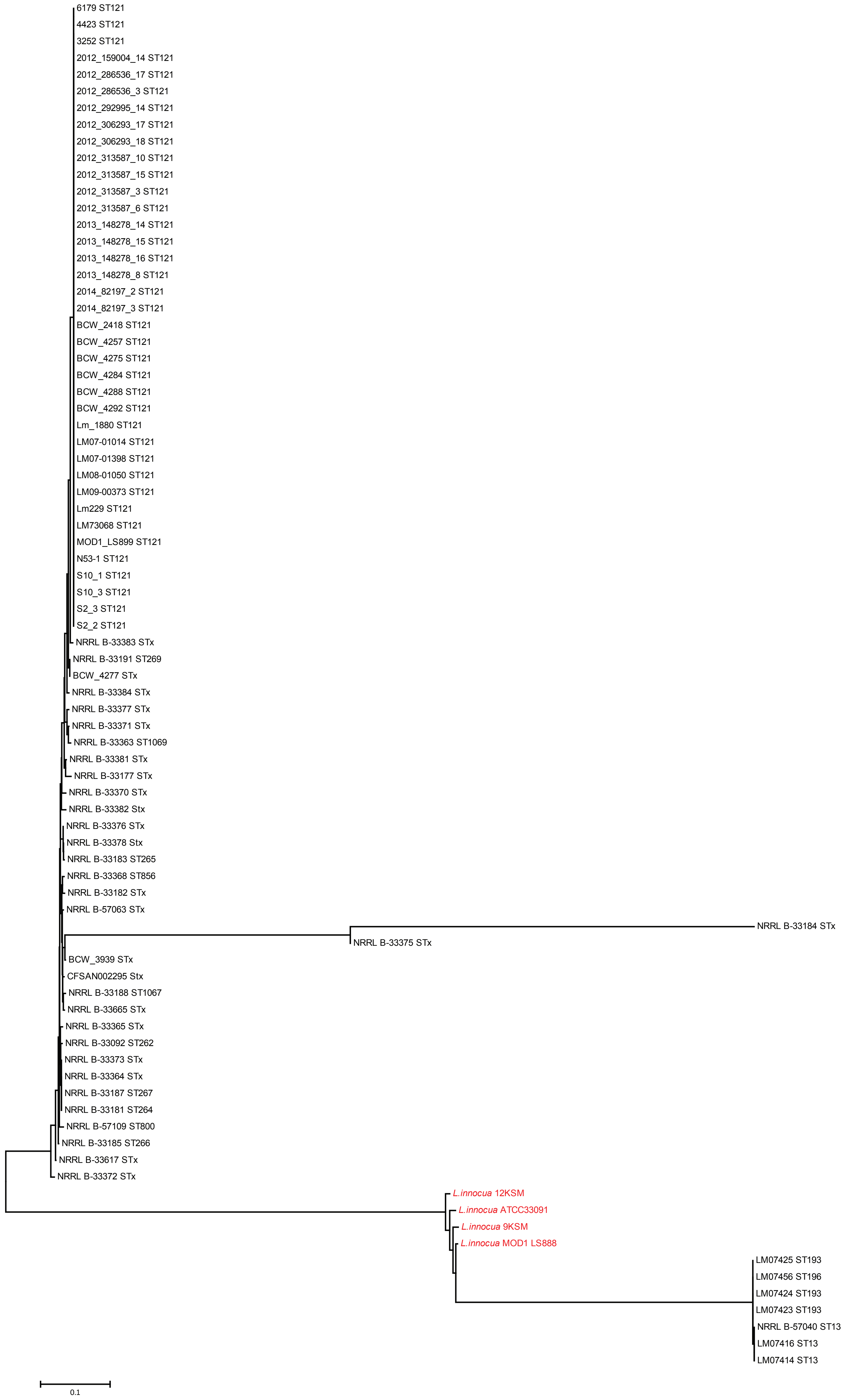

FIG S1 Molecular phylogenetic analysis of SSI-2 by maximum likelihood method based on the Tamura-Nei model. The analysis involved the SSI-2 nucleotide sequence of 78 L. monocytogenes and 4 L. innocua strains (red). There were a total of 1953 positions in the final dataset. STx: unknown ST

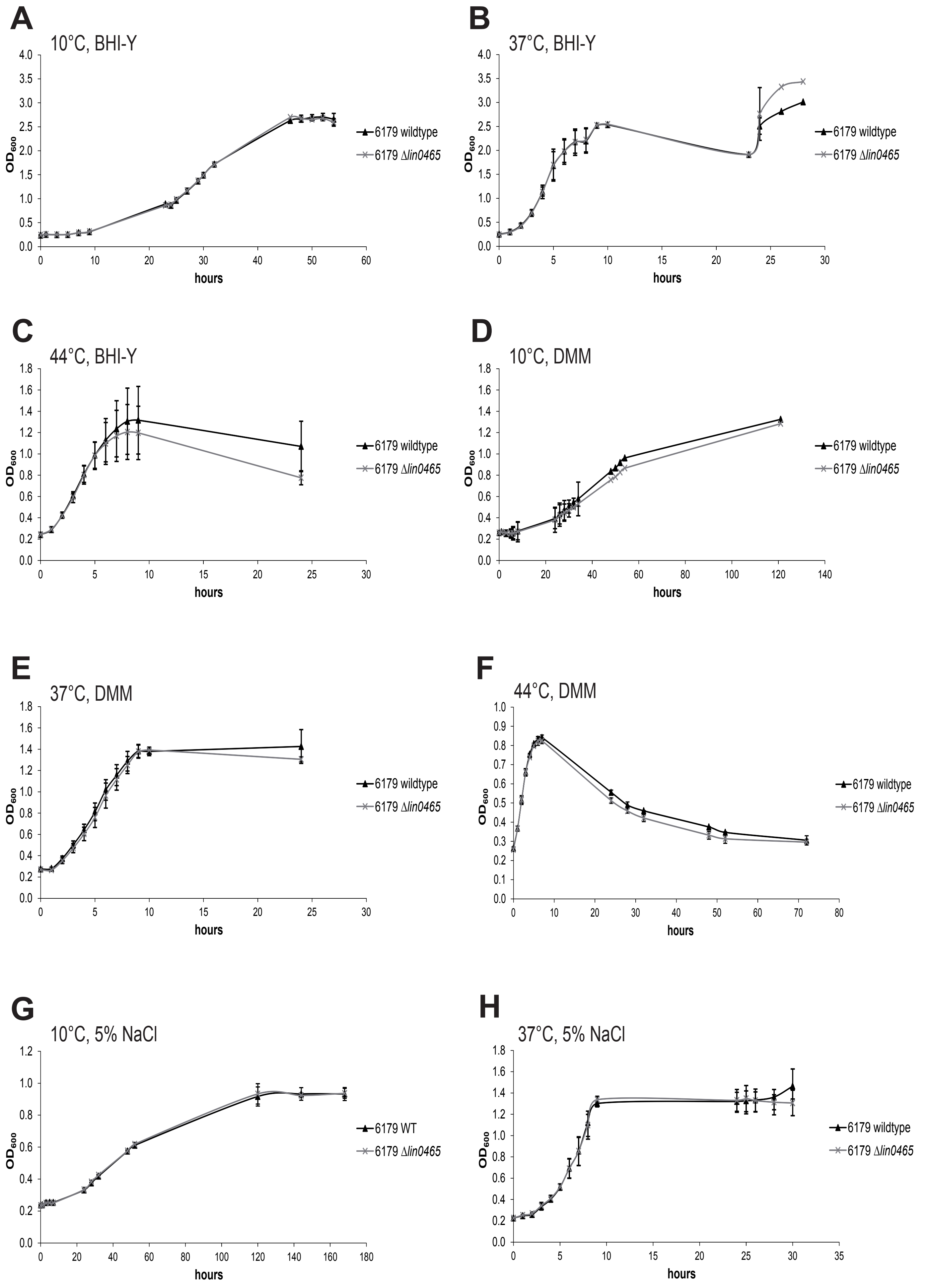

FIG S2 Growth curves for L. monocytogenes 6179 wildtype (WT) and 6179 lin0465 deletion mutant strain (6179 Δlin0465) were established in BHI-Y at 10°C (A), 37°C (B) and 44°C (C), in DMM at 10°C (D), 37°C (E) and 44°C (F) and under osmotic (BHI-Y supplemented with 5% NaCl) at 10°C (G) and 37°C (H). Data represent mean values ± SD of at least three biological replicates.

Table S1: Survival of *L. monocytogenes* 6179 wildtype and *lin0465* deletion mutant strain under acidic, gastric and heat stress.

	mean survival ± SD (%)[a]	
stress condition	6179 wildtype	6179 Δ*lin0465*
pH 2.5, 2 hours	22.73 ± 2.73	23.88 ± 7.42
gastric stress, 2 hours	0.38 ± 0.07	0.34 ± 0.16
55°C, 10 minutes	58.99 ± 11.97	55.34 ± 10.88
55°C, 30 minutes	19.03 ± 8.47	22.03 ± 9.72

[a] Percentage of survival was determined by CFU plate counting. Values represent mean values ± SD of three biological replicates performed in triplicates.

Table S2: Antibiotic susceptibility.

antibiotic compound	disk content	inhibition zone mean ± SD (diameter in mm)	
		6179 wildtype	6179 Δ*lin0465*
benzylpenicillin	1 unit	17.5 ± 0.71	18.0 ± 1.41
cefoxitin	30 µg	11.5 ± 0.71	12.0 ± 1.41
cefoperazone	75 µg	18.0 ± 0.00	18.0 ± 0.00
cefepime	30 µg	8.0 ± 1.41	8.0 ± 1.41
ampicillin	2 µg	21.0 ± 0.00	21.0 ± 1.41
amoxicillin + clavulanic acid	1020 µg	28.5 ± 2.12	29.0 ± 1.41
trimethoprim – sulphamethoxazole	1.25 – 23.75 µg	34.0 ± 0.00	34.0 ± 0.00
gentamycin	10 µg	26.5 ± 0.71	27.0 ± 0.00
norfloxacin	10 µg	20.5 ± 2.12	20.5 ± 0.71
ciprofloxacin	5 µg	21.0 ± 1.41	21.5 ± 0.71
tetracyclin	30 µg	26.0 ± 1.41	26.5 ± 2.12
clindamycin	2 µg	16.0 ± 1.41	16.5 ± 0.71
erythromycin	15 µg	26.5 ± 2.12	26.5 ± 2.12

Table S3: Gene expression of *lin0464* and *sigB* in the *L. monocytogenes* 6179 wildtype, deletion mutant and complemented deletion mutant strain.

**A**	*lin0464* expression (mean ± SD)[a]
6179 wildtype	1.00 ± 0.50
6179 Δ*lin0464*	0.01 ± 0.01
6179 cΔ*lin0464*	668.68 ± 68.16

**B**	*sigB* expression (mean ± SD) [a]
6179 wildtype	1.00 ± 0.23
6179 Δ*sigB*	0.00 ± 0.00
6179 cΔ*sigB*	4.18 ± 0.63

[a]Values were normalized to *16S rRNA* expression levels and are presented as x-fold of the wildtype control. Data represent mean values ± SD of two biological replicates, performed and measured in duplicates.
